# Harnessing Regenerative Science in Aesthetic Surgery: The Biologically Driven Future

**DOI:** 10.3390/jcm14176205

**Published:** 2025-09-02

**Authors:** Claire G. Olivas, Orr Shauly, Dana M. Hutchison, Daniel J. Gould

**Affiliations:** 1 Division of Plastic and Reconstructive Surgery, University of Southern California, Los Angeles, CA 90033, USA; 2 Division of Plastic and Reconstructive Surgery, Emory University, Atlanta, GA 30322, USA; 3 Department of Dermatology, University of California Irvine, Irvine, CA 92617, USA; 4 Private Practice, Beverly Hills, CA 90212, USA

**Keywords:** regenerative medicine, aesthetic surgery, biological therapies, platelet-rich plasma, exosomes, stromal vascular fraction, facial rejuvenation, tissue regeneration

## Abstract

As the fields of plastic surgery and dermatology advance, regenerative medicine is positioned to play a transformative role in both aesthetic and reconstructive procedures. This narrative review examines current and emerging applications of biologic therapies, including exosomes, platelet-rich plasma (PRP), and adipose-derived stem cells (ASCs) with an emphasis on their mechanisms of action, clinical efficacy, and regulatory considerations. We also explore synergistic strategies, such as the combined use of biologics with laser-based technologies, which may enhance therapeutic outcomes. Looking forward, we highlight promising developments in mitochondrial-based therapies, microRNA-based therapies, synthetic exosome mimetics, and AI-assisted biologic design, offering a framework for personalized, precision-driven interventions. By synthesizing existing clinical data alongside scientific and ethical challenges, this narrative review provides a comprehensive perspective on how regenerative therapies are transforming the landscape of aesthetics. Ultimately, successful integration of these innovations will require rigorous validation, ethical responsibility, and a patient-centered approach by plastic surgeons and dermatologists to ensure both safety and accessibility in mainstream practice.

## 1. Introduction

Aesthetic surgery has traditionally relied on techniques that reshape tissue to achieve youthful, harmonious results [1]. Procedures such as facelifts, blepharoplasties, and liposuction have long formed the cornerstone of surgical rejuvenation, with outcomes primarily driven by mechanical manipulation and volume redistribution [2,3,4,5]. While these methods have proven effective, they often address the superficial signs of aging without targeting the underlying biological mechanisms driving tissue degeneration [6,7,8].

In recent years, aesthetic surgery has experienced a paradigm shift toward regenerative science and the integration of biologically active therapies [9]. The growing interest in regenerative medicine reflects a broader movement in medicine toward treatments that restore and enhance the body’s intrinsic healing capabilities [10]. In the context of aesthetics, biologics such as platelet-rich plasma (PRP), exosomes, and autologous fat grafting enriched with stromal vascular fraction (SVF) are being increasingly explored for their potential to promote tissue regeneration and achieve longer-lasting, more natural results [11].

Yet, despite growing enthusiasm, there are gaps and controversies that persist in the literature. Evidence supporting these modalities often consists of small case series, heterogeneous protocols, and variable outcome measures, limiting comparability across studies. Questions remain about standardization of biologic preparations and the balance between regulatory innovation and patient protection. Furthermore, while regenerative aesthetics holds significant potential, the mechanisms of these interventions are not yet fully understood, highlighting the need for translational research and high-quality clinical trials.

This comprehensive narrative review explores the transformative potential of regenerative science in aesthetic surgery. Given the breadth of biologic therapies and the early, heterogeneous nature of the available evidence, a systematic review was not the most appropriate approach. Instead, we undertook a comprehensive narrative review to synthesize emerging findings and contextualize their relevance to aesthetic surgery. We begin by outlining key classes of biologic therapies, then review their mechanisms and clinical applications, and conclude with ongoing challenges and future perspectives in regenerative aesthetics.

## 2. Methods

### 2.1. Literature Search Strategy

A comprehensive literature search was conducted to identify relevant studies and articles related to the role of regenerative medicine in aesthetic surgery. PubMed was searched from its inception up to July 2025. The search terms included a combination of keywords such as “platelet-rich plasma,” “exosomes,” “fractional CO_2_ lasers,” “adipose-derived stem cells,” “stromal vascular fraction,” “facial rejuvenation,” “hair restoration,” “acne scars,” and related terms. Furthermore, a thorough examination of the reference lists of each retrieved article was conducted to identify other relevant information.

### 2.2. Inclusion and Exclusion Criteria

Studies were included if they focused on the application of biologics in aesthetics in one of several domains including but not limited to facial rejuvenation, hand rejuvenation, hair restoration, and acne scars. The inclusion criteria encompassed original research articles, review papers, and case studies.

### 2.3. Data Extraction and Synthesis

Relevant data were extracted from the selected articles, and the extraction process was performed independently by two reviewers (DMH and CGO), and any discrepancies were resolved through consensus (OS). The findings from the selected studies were synthesized to provide a comprehensive overview of the current state of regenerative medicine in aesthetics.

## 3. Overview of Regenerative Medicine

Defined as a cross-disciplinary domain, regenerative medicine centers on the restoration of function through the regeneration of cells, tissues, or organs damaged by congenital anomalies, disease processes, trauma, or the natural aging process [12,13]. Key biologic tools in this field include exosomes, PRP, adipose-derived stem cells (ASCs), and extracellular matrix (ECM)-based scaffolds, which will be further discussed in this paper (Table 1).

### 3.1. Exosomes

**Figure 1:**
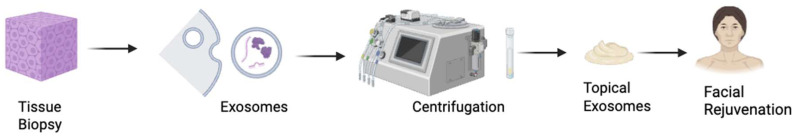
Exosomes’ preparation and mechanism for facial rejuvenation.

Exosomes, a subset of extracellular vesicles (EVs), were first identified in 1983 and initially dismissed as mere cellular waste [14,15,16]. However, subsequent research has revealed their essential role in intercellular communication and signal transduction [17]. These vesicles, typically ranging from 40 to 160 nanometers in diameter, are secreted by various cell types under both physiological and pathological conditions [18]. Their cargo, comprising proteins, lipids, RNA, and DNA, reflects the state of the originating cell and can significantly influence the behavior and function of recipient cells. Growing interest has emerged around leveraging the biological mechanisms of exosomes for therapeutic purposes, particularly their ability to modulate inflammation, stimulate collagen production, and promote tissue repair (Figure 1) [19]. Compared to living stem cells, exosomes offer practical advantages in terms of production and delivery [20]. These properties are especially attractive in aesthetic medicine, where subtle and natural rejuvenation is desired without the risks of surgery. In regenerative medicine, exosomes represent a promising avenue for activating endogenous repair pathways in damaged tissues, potentially addressing conditions that lack effective treatment options [21].

### 3.2. PRP

**Figure 2:**
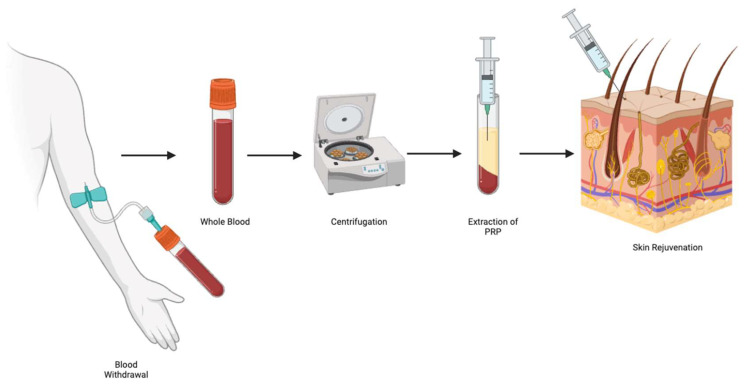
Platelet-rich plasma’s preparation for skin rejuvenation.

PRP was first introduced in 1954 by Kingsley in the field of dentistry to enhance wound healing [22]. Since then, its applications have expanded widely, becoming an increasingly valuable tool in regenerative medicine. PRP is derived from autologous blood and contains a high concentration of platelets suspended in plasma, enriched with growth factors and cytokines [23]. These bioactive molecules are believed to mediate tissue repair, promote angiogenesis, and regulate genes involved in cellular proliferation. Although the precise mechanisms underlying PRP’s effects on facial rejuvenation remain unclear, its ability to modulate the local microenvironment and stimulate endogenous healing processes has led to its growing use in aesthetic medicine, orthopedics, dermatology, and beyond (Figure 2) [24,25].

### 3.3. ASCs

**Figure 3:**
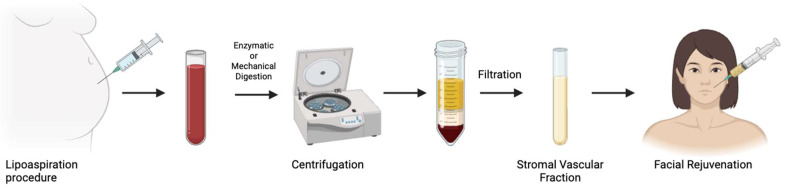
Nanofat preparation for facial rejuvenation.

ASCs, first identified as a distinct mesenchymal stem cell (MSC) population in 2001, have become a cornerstone of regenerative medicine due to the abundance and accessibility of adipose tissue [26]. Residing within specialized “stem cell niches” in adipose tissue, ASCs interact intimately with the ECM and surrounding cells, which modulate their ability to proliferate, migrate, and differentiate key processes for tissue regeneration and wound healing [27]. Beyond their multipotency, ASCs are recognized for their potent paracrine activity, secreting a diverse array of immunomodulatory, angiogenic, and inflammatory cytokines such as interleukin 6 (IL-6), interleukin 8 (IL-8), tumor necrosis factor alpha (TNF-α), as well as growth factors such as platelet-derived growth factor (PDGF), hepatocyte growth factor (HGF), and vascular endothelial growth factor (VEGF) [28,29,30]. These secreted factors promote dermal regeneration and play a critical role in preserving the dermo-epidermal structure in response to local damage. Clinically, ASCs are most applied via autologous fat grafting, a technique celebrated for its biocompatibility and natural aesthetic [31]. Since Neuber’s first use of fat grafting in 1893, lipofilling has evolved into a versatile tool for facial rejuvenation, breast reconstruction, and wound repair [32]. More recently, innovations like nanofat grafting, introduced by Tonnard et al. in 2013, have further expanded the cosmetic applications of ASC-enriched fat, offering non-volumizing regenerative benefits in minimally invasive procedures (Figure 3) [33].

### 3.4. ECM-Based Scaffolds

ECM-based scaffolds, including dermal matrices, recapitulate the architecture and signaling of native ECM, thereby fostering adhesion and expansion of cells alongside active collagen synthesis [34,35]. These bioactive scaffolds aim to guide tissue regeneration by mimicking the natural architecture and signaling cues of healthy tissue. Emerging applications in regenerative medicine include wound healing, scar remodeling, and skin rejuvenation [27,36,37]. By providing both mechanical support and biological signals, ECM-based scaffolds hold significant promise in replacing or augmenting damaged tissue with structurally and functionally optimized alternatives, potentially transforming the landscape of reconstructive and aesthetic surgery [38,39]. Many synthetic scaffolds have now integrated additional cellular signals coated throughout the architecture, to promote additional neovascularity and more directed healing and growth (Table 2).

## 4. Clinical Applications

### 4.1. Non-Surgical Treatments

As patients increasingly seek rejuvenation options with less risk, faster recovery, and natural-looking results, non-surgical therapies have emerged as a cornerstone of modern aesthetic practice [40,41]. Treatments such as exosome-based serums and PRP appeal to patients seeking natural-looking results, as they stimulate the skin’s intrinsic healing processes while avoiding the risks and downtime associated with surgery.

#### 4.1.1. Topical Exosomes

Exosomes have rapidly gained traction in the cosmetic and aesthetic industries, particularly as active ingredients in topical products such as creams, serums, and facial masks [42,43]. These nano-sized extracellular vesicles are loaded with bioactive molecules that promote skin regeneration, hydration, and repair. When applied to the skin, exosomes are believed to stimulate the production of collagen, reduce inflammation, and protect against environmental stressors such as UV radiation and pollution [42,43,44]. Common sources include platelet-derived and MSC-derived exosomes, which are both valued for their regenerative and anti-inflammatory properties in skin repair and rejuvenation.

Beyond their regenerative effects, exosomes show promise in addressing skin pigmentation concerns. By influencing melanogenesis, exosomes may help reduce hyperpigmentation, including dark spots, and uneven skin tone [45,46,47]. Their anti-inflammatory properties may further soothe irritated or inflamed skin, which can also contribute to post-inflammatory hyperpigmentation. Moreover, their ability to be tailored to specific skin concerns opens the door for personalized skincare solutions targeting pigmentation irregularities.

Although the U.S. Food and Drug Administration (FDA) has not approved any injectable exosome therapies for clinical use, many topical formulations are marketed under the cosmetic category, which does not require premarket FDA clearance [48]. This regulatory loophole has enabled rapid commercialization, despite a lack of robust clinical trials validating efficacy and safety [49]. As a result, consumers and clinicians should approach these products with cautious optimism while the scientific and regulatory communities work to catch up with demand.

#### 4.1.2. Injectable PRP

While exosomes represent a novel modality for optimizing the skin’s regenerative microenvironment, PRP continues to be the most widely used biologic [50]. PRP offers several practical advantages: it is autologous, inexpensive, rapidly isolatable, and associated with minimal side effects [51,52,53]. Its regenerative potential derives from concentrated growth factors that promote healing and support paracrine signaling, positioning it as a promising option in aesthetic and regenerative medicine.

Over the past decade, PRP has gained popularity in non-surgical skin rejuvenation. Platelets are a logical candidate for cellular renewal and tissue repair due to their accessibility and abundance of regenerative cytokines [54]. Comparable to exosomes, PRP has shown promise in addressing concerns such as hyperpigmentation, contributing to a more even skin tone and improved complexion [55,56].

However, one of the limitations of PRP lies in its autologous nature. Although this reduces the risk of immune rejection, it also introduces variability based on patient-specific factors. The quality of PRP can decline with age, largely due to reduced concentrations of growth factors [57,58]. This variability underscores the need for further optimization and standardization to ensure consistent therapeutic outcomes across patient populations.

#### 4.1.3. Exosomes Compared to PRP

The use of exosomes represents a novel approach in aesthetic medicine, offering outcomes that may be comparable or even superior to those achieved with PRP for skin rejuvenation, without the need for blood draws. In a study by Estupiñan et al., exosomes used in conjunction with radiofrequency microneedling achieved similar or improved results compared to PRP in enhancing skin texture and tone [59].

From the patient perspective, exosomes offer a less invasive alternative, which may be especially appealing to individuals who are needle-averse or prone to vasovagal reactions. Furthermore, unlike PRP, which requires processing the patient’s blood, exosome preparations are immediately available for use, potentially reducing procedure times. A summary of the advantages and disadvantages of exosomes, PRP, and other regenerative therapies is presented in Table 3.

### 4.2. Surgical Treatments

While non-surgical aesthetic treatments continue to rise in popularity, certain surgical interventions remain highly sought after for their transformative and long-lasting results. Among these, enriched fat grafting supplemented with stromal vascular fraction (SVF) has garnered significant interest from plastic surgeons, dermatologists, and patients. This technique not only restores volume but also promotes tissue regeneration and dramatically enhances skin quality, making it a compelling option in regenerative aesthetic surgery.

#### 4.2.1. Enriched Fat Grafting

ASC-enriched fat grafting has gained popularity in facial rejuvenation, particularly since Tonnard et al. introduced the concept of nanofat in 2013. Clinically, it is important to differentiate between microfat, nanofat, and SVF-enriched fat. Microfat consists of intact adipocytes harvested via liposuction and is used primarily for restoration of volume due to its structural integrity. Nanofat is produced by emulsifying and filtering microfat harvested via liposuction, yielding a whitish fluid composed of approximately 25% adipocytes and a 75% SVF [60]. This SVF contains a rich mix of regenerative cells, including ASCs, endothelial cells, monocytes/macrophages, granulocytes, and lymphocytes [61]. Nanofat grafting offers a compelling and natural-looking alternative to synthetic fillers, with a potential for longer-lasting results [62]. SVF-enriched fat grafting combines microfat with concentrated SVF to enhance both graft survival and regenerative effects, making it useful in compromised tissue. These biologic approaches have demonstrated multi-targeted regenerative effects, prompting increased interest in both preclinical and clinical settings. Applications span a wide range of conditions, including atopic dermatitis (AD), vitiligo, psoriasis, acne, lichen sclerosus (LS), chronic wounds, and alopecia [63].

Nevertheless, several limitations must be acknowledged, such as donor site morbidity, variability in graft survival, risk of graft hypertrophy, and the need for local or general anesthesia [64].

#### 4.2.2. Laser + Biologics Synergy

Laser technologies have significantly advanced the field of skin rejuvenation, offering minimally invasive solutions that reduce recovery time while achieving outcomes previously thought unattainable [65,66]. Low-level laser therapy (LLLT) has demonstrated the ability to enhance mitochondrial function, boost ATP production, and reduce oxidative stress conditions that are essential for optimizing stem cell viability [67]. These favorable effects position laser therapy as a promising adjunct in wound care, enhancing healing, reducing pain, and improving outcomes in dermatologic and reconstructive conditions [68].

When combined with biologics, lasers may further amplify regenerative outcomes. For instance, pairing laser therapy with PRP has been shown to enhance skin texture, reduce wrinkles, and improve acne scars, while also mitigating laser-induced trauma [69]. Similarly, the use of laser-assisted nanofat grafting may expedite tissue regeneration and wound healing, offering synergistic benefits and potentially reducing adverse effects [70]. In the context of exosome therapy, laser preconditioning may enhance cellular uptake and improve therapeutic delivery, making this approach an emerging option in aesthetic and regenerative medicine. Nevertheless, limitations remain, including the frequent need for multiple treatment sessions, the sensitivity of outcomes to baseline skin tone, and the risk of both hypo- and hyperpigmentation, all of which warrant cautious patient selection and counseling.

## 5. Clinical Evidence

To fully appreciate the translational potential of regenerative therapies in aesthetic medicine, it is critical to evaluate the clinical evidence supporting their use. This review evaluates the efficacy, safety, and patient-reported outcomes of four key modalities: exosomes, PRP, ASCs, and laser–biologic combinations (Table 4).

### 5.1. Clinical Evidence—PRP

PRP has been clinically evaluated across a variety of aesthetic indications, including facial rejuvenation, hand rejuvenation, hair restoration, and acne scarring. In a randomized clinical trial on facial rejuvenation, Alam et al. reported significant improvement in skin texture and wrinkle severity on the PRP-treated side of the face, as assessed by both patients and investigators [71]. Cabrera-Ramírez et al. investigated PRP for photodamaged dorsal hand skin and observed histologic increases in fibroblast proliferation and neovascularization in the superficial dermis [72]. In the context of androgenic alopecia, Qu et al. demonstrated that PRP enhanced hair regrowth and promoted sustained effects across multiple hair cycles by modulating the hair follicle environment [73]. Similarly, Gulanikar et al. explored the efficacy of PRP in treating acne scars, noting that 50% of patients with Grade 2–3 scars improved, and among those with Grade 3 scars, 60% exhibited improvement by at least one grade. Importantly, patient-reported outcomes paralleled these clinical findings: over half of participants rated their satisfaction as “very good” and the remainder as “good” on a four-point scale [74]. Compared with other biologic therapies, the evidence base for PRP is relatively robust, supported by multiple randomized controlled trials, though inconsistent preparation protocols hinder standardization and broader adoption.

### 5.2. Clinical Evidence—Exosomes

Exosomes have emerged as a promising alternative in regenerative aesthetics, with clinical studies evaluating their efficacy in facial rejuvenation, hair restoration, and acne scar management. Although research is still preliminary and widespread clinical application remains distant, initial findings highlight their therapeutic potential. In a study by Proffer et al., topical application of platelet-derived exosomes resulted in marked improvements in overall skin quality, including reductions in redness, wrinkle depth, and melanin content across multiple facial aesthetic units. Participant-reported outcomes further supported these findings, with positive responses at six weeks and 98.2% of patients expressing willingness to continue treatment [76]. Additionally, Kim et al. examined the role of exosomes in non-scarring alopecia and found that they promoted hair regeneration by facilitating the telogen-to-anagen transition through activation of the Wnt/β-catenin signaling pathway [77].

### 5.3. Clinical Evidence—ASCs

ASC-based therapies have shown clinical utility in facial rejuvenation, hair restoration, and scar remodeling. Yao et al. reported that SVF gel improved lower eyelid hyperpigmentation and periorbital wrinkles, highlighting its role in skin rejuvenation. Beyond objective measures, patient perceptions further underscored the advantage of SVF gel: on a 5-point Likert scale, 77.3% of patients reported being satisfied (54.5%) or very satisfied (22.8%) with their outcomes, compared with 53.8% satisfaction (48.7% satisfied; 5.1% very satisfied) in the conventional lipoinjection group [79]. In a study on hair loss, Kim et al. found that SVF gel significantly increased hair density and keratin content, suggesting improved follicular health and function [80]. For acne scars, Behrangi et al. demonstrated that SVF injections led to significant improvements in scar volume, surface area, and depth, alongside increases in dermal and epidermal thickness [81]. While these findings are encouraging, the current evidence remains largely preliminary and derived from small-scale or early-phase clinical studies, demonstrating the need for larger randomized controlled trials to validate safety and efficacy.

### 5.4. Clinical Evidence—Lasers and Biologics

The synergistic use of laser technology with regenerative biologics, such as PRP, exosomes, and nanofat, has been explored in the treatment of facial aging and acne scarring. This combined approach seeks to harness the ablative or fractional effects of lasers to enhance the penetration and efficacy of biologic agents. Rageh et al. evaluated the combination of fractional CO_2_ lasers with nanofat in patients with acne scars and reported a significant reduction in Goodman and Baron quantitative acne scar scores following treatment, demonstrating both safety and efficacy [82]. Similarly, Kwon et al. investigated the combined application of CO_2_ lasers and exosomes, finding that exosome-treated areas showed significantly greater improvements in scar quality compared to control-treated regions [70]. In a separate study, Dayan et al. examined the adjuvant effect of topical exosomes following CO_2_ laser resurfacing and observed enhanced improvements in skin brightness and overall youthful appearance by days 14 and 30 post-treatment [78]. The synergistic benefits of PRP and laser therapy were also explored. Hui et al. studied the effects of CO_2_ laser treatment combined with intradermal PRP in facial rejuvenation and found that the combination significantly improved skin texture, elasticity, and wrinkle severity while minimizing post-treatment downtime and adverse effects. Satisfaction rates were higher in the combination group compared with controls for facial wrinkles (76.9% vs. 69.2%), skin texture (84.6% vs. 76.9%), and skin elasticity (69.2% vs. 61.5%), with differences reaching statistical significance [75]. Nevertheless, as these findings are derived from experimental studies, laser–biologic therapies should be regarded as promising yet still investigational within practice.

### 5.5. Limitations

As enthusiasm for regenerative medicine continues to grow, it is imperative that the medical community, regulatory bodies, and bioethicists work in concert to ensure that emerging therapies are developed and implemented with the patient’s best interests at the forefront [83]. Achieving this requires transparency, with patients fully informed of both the experimental status and the regulatory limitations of biologic therapies currently in use. However, this is no different than the ethical obligation that has always guided medical innovation, ensuring that patients understand the balance between potential benefit and risk, and that clinical progress is pursued without compromising safety, integrity, or trust.

Across the spectrum of regenerative modalities, including exosomes, PRP, ASCs, and laser–biologic combination therapies, there remains a critical lack of high-quality, large-scale clinical trials validating safety, efficacy, and long-term outcomes. Additionally, regulatory oversight is still evolving [84]. For example, exosome-based products have not been approved for clinical use by the FDA, EMA, or MHRA, all of which have issued warnings highlighting the absence of sufficient evidence and standardized production practices [85]. Despite these warnings, unapproved exosome formulations, including plant-derived and animal-derived sources, are being promoted online and administered in aesthetic clinics across North America and Europe, often without peer-reviewed data or ethical sourcing practices [86].

Similar concerns apply to PRP- and ASC-based therapies. These autologous products often fall into regulatory gray zones, where lack of standardization in preparation, concentration, and administration can lead to variable therapeutic outcomes [87]. Furthermore, the use of lasers with biologics lacks consistent clinical protocols, making comparative evaluation and reproducibility challenging. In addition to regulatory gaps, biologic products are inherently subject to batch-to-batch variability, complex isolation procedures, and challenges in large-scale manufacturing [88]. For extracellular vesicles and nanofat-derived therapies, therapeutic efficacy often hinges on the bioactivity of the cellular cargo, which is sensitive to donor variability and processing conditions.

Ethical considerations are also emerging, as patients grow more conscious of treatments’ origins, particularly in relation to sustainability and sourcing [89,90]. This may influence future preferences for plant-based or synthetic biologics, provided they can demonstrate comparable safety and effectiveness.

Addressing these limitations through rigorous clinical investigation, standardized manufacturing practices, and clearer regulatory frameworks will be essential to responsibly advance the field of regenerative aesthetics.

## 6. Regulatory and Ethical Considerations

As aesthetic surgery increasingly embraces longevity-focused interventions and biologically driven therapies, the ethical and regulatory landscape must evolve accordingly. A central concern in the use of biologics is the risk associated with non-compliant product use. Off-label application without proper documentation, informed consent, or clinical justification may expose practitioners to legal liability. Additionally, improper handling and lack of sterility can result in infection, immune reactions, inflammatory complications, or inconsistent therapeutic outcomes, particularly in a field where standardized protocols remain limited.

A growing number of biologic products, such as exosome-based treatments, are being marketed for aesthetic use without FDA approval or sufficient clinical validation, placing patients at risk and eroding public trust [91]. Addressing this issue requires a concerted effort to educate patients and providers alike. Physicians should maintain transparency about which treatments are FDA-approved, what clinical evidence supports their use, and which products remain experimental or unregulated. Large Language Models (LLMs), including tools like ChatGPT 5.0, may offer a promising adjunct by generating comprehensible educational content that supports informed decision-making [92,93].

To ethically advance regenerative aesthetics, it is imperative to establish standardized methods for biologics’ isolation, characterization, and application. Rigorous clinical trials and active collaboration with regulatory agencies will be essential to create enforceable guidelines that safeguard patient safety while fostering responsible innovation.

## 7. The Future of Regenerative Aesthetics

As regenerative aesthetics progresses beyond foundational therapies like PRP and ASCs, the field is rapidly advancing toward next-generation technologies that enable highly personalized interventions. Innovations such as mitochondrial-based therapies, microRNA (miRNA)--based therapies, synthetic exosome mimetics, and AI-assisted development of biologics are redefining the role of biologics in aesthetic medicine (Table 5).

### 7.1. Mitochondria

Mitochondria play a pivotal role in cellular aging, regulating energy metabolism, oxidative stress, and skin regeneration. Dysregulation of mitochondrial function has been linked to the degradation of dermal collagen and elastin, contributing to visible signs of aging [94]. As regenerative aesthetics moves toward more targeted interventions, mitochondria-based therapies are gaining interest for their potential to restore skin vitality from within. Emerging strategies include mitochondrial-targeted antioxidants, NAD^+^ precursors, and autophagy-activating compounds, each designed to optimize mitochondrial performance [95,96]. These approaches may complement or enhance existing biologic treatments by addressing the energetic and metabolic deficits underlying tissue degeneration. Moreover, preserving mitochondrial integrity has been associated with improved skin barrier function and prolonged cellular longevity [97]. While early data is promising, mitochondrial therapies remain largely experimental. Further research is essential to determine their long-term clinical safety and efficacy in aesthetic applications [98,99].

### 7.2. MicroRNA-Based Therapies

miRNA-based therapies represent a novel approach in regenerative aesthetics, enabling reversible control of gene expression linked to skin’s aging and inflammation [100]. By regulating post-transcriptional gene regulation, miRNAs suppress collagen degradation and inflammation, promoting skin repair and rejuvenation [101]. Specific miRNAs with anti-inflammatory properties have shown promise treating conditions like psoriasis, atopic dermatitis, and cutaneous wounds [102]. In addition, miRNAs also serve as biomarkers for diagnosis and prognosis, offering a personalized approach to aesthetic care. Despite challenges with delivery, stability, and cost, ongoing research is advancing toward clinical translation [103].

### 7.3. Synthetic Exosome Mimetics

Exosomes are attractive in regenerative medicine for their biocompatibility, low immunogenicity, and ability to cross biological barriers [104]. However, their clinical use is limited by low yields and heterogeneity. To address these challenges, synthetic exosome mimetics have been engineered to replicate the structure and function of natural exosomes [105].

Resembling liposomes or native exosomes, biomimetic exosomes exhibit enhanced biocompatibility, chemical stability, cost-effectiveness, and scalability when compared with conventional lipid nanoparticles. Their synthetic nature also allows for large-scale production, with greater control over cargo and composition [106].

Despite these advantages, clinical application remains in its infancy. Barriers include the lack of standardized protocols, as well as the need for fully automated purification systems [105]. Nonetheless, preclinical and early clinical studies are promising. For instance, Kim et al. reported that synthetic exosomes enhanced skin barrier repair and stimulated angiogenesis in atopic dermatitis, offering an early example of their regenerative potential in dermatologic and aesthetic contexts [107].

### 7.4. AI-Assisted Biologics

Artificial intelligence (AI) is becoming a powerful tool in regenerative aesthetics, particularly in optimizing the development and application of biologics. One promising area is scaffold design, where AI algorithms can analyze material properties, simulate fabrication processes, and predict functional outcomes to guide optimization [108]. These processes also enhance quality control by monitoring production in real time and detecting deviations from established parameters. Beyond scaffolds, AI helps identify optimal cellular sources for biologic therapies, such as exosomes or stem cell-derived products, based on desired regenerative outcomes. AI-driven approaches are expected to streamline discovery and commercialization of next-generation regenerative treatments, marking a new era of efficiency in aesthetic medicine [109].

## 8. Conclusions

With the evolution of aesthetic surgery, regenerative medicine will shift from an optional tool of the surgical repertoire to becoming integral to modern practice. By addressing the biological root causes of aging and tissue degeneration, regenerative therapies offer natural, durable, and high-quality solutions that enhance both aesthetic outcomes and long-term tissue health. As these innovations become increasingly integrated into clinical care, plastic surgeons and dermatologists must lead the way, not only in applying these therapies but in researching their safety, efficacy, and long-term outcomes. Establishing clear ethical and clinical guidelines will be essential to ensuring that innovation progresses responsibly and with patients’ best interests in mind.

## Figures and Tables

**Table 1:** Summary of regenerative medicine modalities. PRP: platelet-rich plasma; ASCs: adipose-derived stem cells; ECM: extracellular matrix.

Regenerative Product Type	Mechanism of Action	Summary of Clinical Indications
PRP	Concentrated release growth factors that stimulate fibroblast proliferation, angiogenesis, and extracellular matrix ECM remodeling.	Facial rejuvenation (wrinkles, texture, elasticity) Hand rejuvenation Chronic wound healing Scar revision Acne scars Alopecia/hair restoration Pigmentary disorders
Exosomes	Nanovesicles secreted by cells containing proteins, lipids, and miRNAs. Modulate intercellular communication, reduce inflammation, promote angiogenesis, and stimulate fibroblast and keratinocyte activity.	Facial rejuvenation (wrinkles, texture, elasticity) Chronic wound healing Scar revision Alopecia/hair restoration
ASCs	Multipotent mesenchymal stem cells secrete cytokines and growth factors with paracrine regenerative effects. Enhance angiogenesis, modulate immune response, and promote adipogenesis and collagen remodeling.	Facial rejuvenation (wrinkles, texture, elasticity) Hand rejuvenation Chronic wound healing Scar revision Acne scars Alopecia/hair restoration Radiation-induced damage
ECM-Based Scaffolds	Decellularized extracellular matrices retain structural proteins and bioactive molecules that mimic native tissue architecture. They facilitate cell adhesion, migration, and proliferation; modulate immune responses; and promote angiogenesis.	Facial rejuvenation (wrinkles, texture, elasticity) Chronic wound healing Scar revision Soft tissue augmentation

**Table 2:** Role of growth factors in regenerative medicine. PDGF: platelet-derived growth factor; HGF: hepatocyte growth factor; VEGF: vascular endothelial growth factor; IL-6: interleukin 6, IL-8: interleukin 8; TNF-α: tumor necrosis factor alpha.

Growth Factor	Role
PDGF	Stimulates fibroblasts, collagen, and angiogenesis
VEGF	Promotes new blood vessels’ formation
HGF	Enhances cell migration and supports stem cell-mediated tissue regeneration
Inflammatory cytokines (IL-6, IL-8, TNF-α)	Regulate inflammation and initiate tissue repair

**Table 3:** Advantages and disadvantages of different types of regenerative medicine modalities.

Regenerative Product Type	Advantages	Disadvantages
PRP	Minimal discomfort in harvesting More cost-effective	Lack of standardization Limited depth of tissue penetration
Exosomes	Lower immunogenicity Potent paracrine signaling	Difficult to assess exosome content Regulatory issues
ASCs	Abundant source Multipotent, with regenerative potential	Requires liposuction and processing Viability and yield can vary
Biologics/Lasers	Enhanced collagen remodeling Maximizes skin-rejuvenating effects	Increased cost Greater operator dependence

**Table 4:** Key studies of regenerative therapies with diverse clinical applications. SVF: stromal vascular fraction.

Study	Level of Evidence	Product Type	Intended Effect	Efficacy	Safety
Alam et al., 2018 [71]	Experimental study/II	PRP	Facial Rejuvenation	Skin treated with platelet-rich plasma was found to be significantly less rough and wrinkled at 6 months.	No side effects associated with PRP directly.
Cabrera-Ramírez et al., 2017 [72]	Experimental study/II	PRP	Hand Rejuvenation	Histological analysis showed an increase in the number of fibroblasts (*p* < 0.001), number of vessels (*p* < 0.001), and collagen density (*p* = 0.27).	No side effects reported.
Qu et al., 2021 [73]	Experimental study/II	PRP	Hair Restoration	Significant increase in hair density, hair count, diameter, and anagen hair ratio at 6 months compared to the control side and baseline.	No side effects reported.
Gulanikar et al., 2019 [74]	Case series/IV	PRP	Acne Scars	All the types of scars showed response in terms of reduction in size.	Mild erythema and edema lasting for 1 day.
Hui et al., 2017 [75]	Experimental study/II	CO_2_ lasers + PRP	Facial Rejuvenation	Combination treatment of PRP and laser was superior to laser treatment alone. Scores reflect better area and density of wrinkles, texture, and pores.	Mild erythema, edema, and crusting in both the experimental group and control group, with less side effects reported in the experimental group.
Proffer et al., 2022 [76]	Case series/IV	Exosomes	Facial Rejuvenation	VISIA-CR imaging yielded quantifiable and statistically significant improvements in overall skin health.	Mild dryness, with no other side effects reported.
Kim et al., 2022 [77]	Preclinical study/V	Exosomes	Hair Restoration	Exosomes induced proliferation of DP cells and accelerated hair regeneration through activation of the Wnt/β-catenin pathway.	No side effects reported.
Kwon et al., 2020 [70]	Experimental study/II	CO_2_ lasers + Exosomes	Acne Scars	Atrophic scar volume, mean pore volume, and skin surface roughness were significantly decreased from baseline on the ASC side.	Mild erythema, post-treatment pain, and dryness that resolved within 5 days.
Dayan et al., 2023 [78]	Experimental study/II	CO_2_ lasers + Exosomes	Facial Rejuvenation	Statistically significant brighter appearing skin at 14 days and more youthful looking skin on days 14 and 30.	Mild bruising and itching.
Yao et al., 2018 [79]	Cohort study/IV	Nanofat	Facial Rejuvenation	Assessment of patient-rated satisfaction on a 5-point Likert scale found that 77.3% of patients in the SVF gel group were satisfied (54.5%) or very satisfied (22.8%) with their outcomes.	Patients in the SVF gel group experienced mild postoperative swelling.
Kim et al., 2021 [80]	Experimental study/II	SVF	Hair Restoration	Hair density of the SVF-treated side was significantly increased after 3 and 6 months of transplantation compared to the non-treated side.	No side effects reported.
Behrangi et al., 2022 [81]	Experimental study/II	SVF	Acne Scars	The use of SVF in the treatment of patients with acne scars accelerates improvement in volume, area, and depth of the scar.	No side effects reported.
Rageh et al., 2023 [82]	Experimental study/II	CO_2_ lasers + Nanofat	Acne Scars	After treatment, the nanofat treated side of the face showed a significant reduction in Goodman scores.	Erythema, edema, and crust formation were reported by all patients, which faded away within 6.68 ± 0.95 days after the session.

**Table 5:** Comparative overview of next-generation regenerative therapies. ROS: Reactive Oxygen Species.

Technology	Primary Mechanism	Advantages	Limitations
Mitochondrial Therapies	Boost energy metabolism, reduce ROS	Cellular rejuvenation at source, metabolic enhancement	Still experimental; limited human data
MicroRNA Therapies	Post-transcriptional gene regulation	Precision targeting, reversible effects	Delivery challenges, cost
Synthetic Exosome Mimetics	Biologically inspired delivery systems	Scalable, customizable, reduced immune response	Lack of standardization; early-stage clinical use
AI-Assisted Biologics	Data-driven design and optimization	Speeds R&D, enhances personalization	Requires robust datasets, limited clinical integration

## Data Availability

All data are available upon reasonable request from the corresponding author.

## Abbreviations

The following abbreviations are used in this manuscript:

PRP	Platelet-Rich Plasma
ECM	Extracellular Matrix
ASCs	Adipose-Derived Stem Cells
SVF	Stromal Vascular Fraction
LLLT	Low-Level Laser Therapy
AD	Atopic Dermatitis
LS	Lichen Sclerosus
FDA	Food and Drug Administration
EMA	European Medicine Agency
MHRA	Medicines and Healthcare products Regulatory Agency
miRNA	microRNA
LLMs	Large Language Models
ROS	Reactive Oxygen Species
